# Kupffer cells are central in the removal of nanoparticles from the organism

**DOI:** 10.1186/1743-8977-4-10

**Published:** 2007-10-19

**Authors:** Evaldas Sadauskas, Håkan Wallin, Meredin Stoltenberg, Ulla Vogel, Peter Doering, Agnete Larsen, Gorm Danscher

**Affiliations:** 1 Department of Neurobiology, Institute of Anatomy, University of Aarhus, Building 1233/1234, Wilhelm Meyers Allé, DK-8000 Aarhus C, Denmark; 2 National Research Centre for the Working Environment, Lerso Parkalle 105, DK-2100 Copenhagen Ø, Denmark

## Abstract

**Background:**

The study aims at revealing the fate of nanoparticles administered intravenously and intraperitoneally to adult female mice, some of which were pregnant. Gold nanoparticles were chosen as a model because these particles have been found to be chemically inert and at the same time are easily traced by autometallography (AMG) at both ultrastructural and light microscopic levels.

**Results:**

Gold nanoparticles were injected intravenously (IV) or intraperitoneally (IP) and traced after 1, 4 or 24 hours. For IV injections 2 and 40 nm particles were used; for IP injections 40 nm particles only. The injected nanoparticles were found in macrophages only, and at moderate exposure primarily in the Kupffer cells in the liver. IV injections resulted in a rapid accumulation/clustering of nanoparticles in these liver macrophages, while the uptake in spleen macrophages was moderate. IP injections were followed by a delayed uptake in the liver and included a moderate uptake in macrophages located in mesenteric lymph nodes, spleen and small intestine. Ultrastructurally, the AMG silver enhanced nanocrystals were found in lysosome-like organelles of the Kupffer cells and other macrophages wherever located.

Accumulations of gold nanoparticles were not found in any other organs analysed, i.e. kidneys, brain, lungs, adrenals, ovaries, placenta, and fetal liver, and the control animals were all void of AMG staining.

**Conclusion:**

Our results suggest that: (1) inert gold nanoparticles do not penetrate cell membranes by non-endocytotic mechanisms, but are rather taken up by endocytosis; (2) gold nanoparticles, independent of size, are taken up primarily by Kupffer cells in the liver and secondarily by macrophages in other places; (3) gold nanoparticles do not seem to penetrate the placenta barrier; (4) the blood-brain barrier seems to protect the central nervous system from gold nanoparticles; (5) 2 nanometer gold particles seem to be removed not only by endocytosis by macrophages, and we hypothesize that part of these tiny nanoparticles are released into the urine as a result of simple filtration in the renal glomeruli.

## Background

Nanotechnology is a rapidly developing field, and new nanomaterials are daily introduced in new products within electronics, foods, food containers, pharmaceutical drugs, cosmetics, paints and surface coatings [1]. This trend will lead to an ever-increasing presence of nanoparticles in the environment. Therefore, serious considerations have to be made as to whether such particles are harmful to life in general and man in particular. Concerning nanoparticles with high stability one might worry about the consequences of introducing matters with such an immense surface area. Some metals that are otherwise harmless, e.g. metallic zinc, become flammable, if pulverized into micro- and nanosize particles. It is also known that even a moderate exposure to such particulate matters can cause damage to the organism [2]. Another concern is that nanoparticles might penetrate epithelia in the lungs, gastrointestinal tract, and skin and thereby spread in the whole body [1,3,4].

In the present study we evaluated the biodistribution of colloidal gold nanoparticles. Gold nanoparticles are being used for diagnostics, therapy, research etc. [5-8]. With the histochemical technique autometallography (AMG), gold nanoparticles can be traced in morphologically intact tissue [5,9-11]. As a model for evaluation of whether nanoparticles penetrate membranes if introduced parenterally, we injected mice IV and IP with gold nanoparticles, sized 2 and 40 nm, respectively, and looked for them in different organs including brain, lungs, liver, spleen, kidneys, adrenals, ovaries, and small intestine. A group of pregnant animals was included in order to evaluate reveal whether gold nanoparticles penetrate the placenta barrier.

## Results

**Figure 1:**
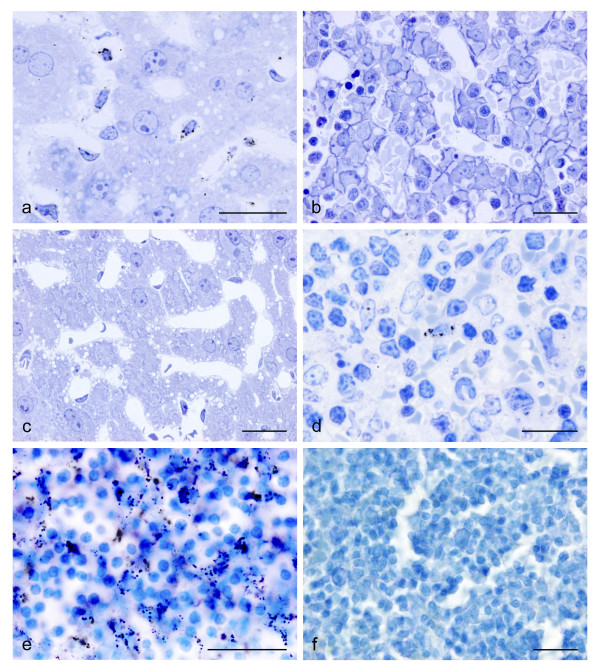
Micrographs demonstrating AMG silver enhanced clustered gold nanoparticles in mouse after in vivo exposure. (a) 40 nm gold particles clustered in Kupffer cells of the liver from a pregnant mouse. (b) Section from a fetal liver taken from an embryo of the same animal. Note that the fetal tissue is completely void of staining. The pregnant animal was intravenously injected 40 nm gold particles and allowed to survive for 24 hours. Both sections were 3 micron Epon sections counterstained with toluidine blue. (c) Section from the liver of a pregnant mouse, which served as a control and was exposed to saline. The section is completely void of AMG staining; (d) Micrograph of nanogold particles in a spleen macrophage. The animal was treated intravenously with 40 nm gold particles 24 hours before being sacrificed. 3 micron Epon section counterstained with Toluidine blue. (e) Enhanced gold nanoparticles in macrophages of a mesenterial lymph node. The animal was injected 40 nm gold nanoparticles intraperitoneally and allowed to survive for 4 hours, 30 μm thick cryo section, counterstained with toluidine blue. (f) Micrograph of a mesenterial lymph node of a mouse which was exposed to saline intraperitoneally and served as control. Scalebars = 20 μm.

In all mice exposed to gold nanoparticles accumulations of nanoparticles were traced in Kupffer cells, i.e. in the macrophages of the liver (Fig. 1a). No accumulations in other cells than macrophages were seen in any of the organs examined in this study and sections from the control animals were all void of AMG staining (Fig. 1c).

The fastest and most intense uptake was observed in Kupffer cells from animals exposed IV to 40 nm nanoparticles. All the Kupffer cells were loaded. In the animals injected IV with 2 nm nanoparticles, the load of AMG silver enhanced nanoparticles was much less intense, and was seen only in a fraction of the Kupffer cells of the liver and some macrophages of the spleen. Animals given 40 nm nanoparticles IP showed far less intense staining of the Kupffer cells and the number of loaded macrophages was profoundly reduced. In these animals a moderate uptake in the macrophages of mesenterial lymph nodes (Fig. 1e) and in the lymphatic tissue in the wall of the small intestine and in the spleen (Fig. 1d) were seen as well.

**Figure 2:**
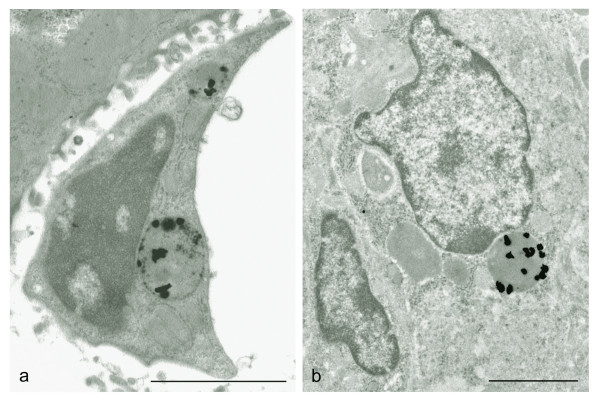
Electron micrographs showing AMG enhanced clustered gold nanoparticles in the lysosomes of a Kupffer cell (a) and a spleen macrophage (b). The animal was exposed to 40 nm gold nanoparticles intravenously and allowed to survive for 24 hours. Scalebars = 2 μm.

Independent of exposure, the gold nanoparticles were found ultrastructurally to accumulate in lysosome-like structures of the macrophages supporting the notion that nanoparticles are taken up by endocytosis (Fig. 2). Careful scanning of AMG silver enhanced sections from kidneys, brain, lungs, adrenals and ovaries of both IP and IV injected animals did not reveal accumulation of nanoparticles in these organs.

### Transplacental transport

Pregnant mice injected IV revealed particle accumulation in the liver of the dams as described above (Fig. 1a). The mice were injected nanoparticles at days 16–18 of pregnancy and killed 24 hours after the last exposure by transcardial perfusion with glutaraldehyde. Sections from placentas and fetuses were AMG silver enhanced in order to trace a possible entrance of gold nanoparticles. No particles were found in the fetuses (Fig. 1b) or in the placentas.

## Discussion

Gold nanocrystals will be silver enhanced if exposed to an autometallographic developer. The AMG technique is based on the catalytic qualities of gold nanoparticles causing silver ions adhering to their surfaces to be reduced to metallic silver atoms powered by electrons released from reducing molecules [9,11]. Some few other metal containing nanocrystals have the same quality making it imperative to include tissue from untreated animals in the analysis. The only other pure metal nanoparticle known to be AMG amplifiable is made of silver atoms. However, mercury, bismuth and silver have been proved to be metabolized into metal – sulphur respectively metal – selenium nanoparticles when introduced in organisms. These nanoparticles can also be enhanced by AMG [11]. The finding that gold nanoparticles can be traced by autometallography was presented in 1981 [9] and three years later the technique was implemented as a tool for tracing RNA and antigens with gold tagged RNA-ase and antibodies [5,11,12]. The amplification power of AMG is substantial – e.g. a 14 nanometer gold nanoparticle will be silver amplified 3,5 times if placed in a silver lactate developer for 15 minutes. A single particle can be detected by AMG even if it is just a fraction of a nanometer in diameter [11,13]. Therefore all the 2 and 40 nanometer gold nanoparticles present in the analyzed tissue will be AMG silver enhanced. Today, the technique is widely used for multitude of purposes and AMG developers are commercially available as are different molecules tagged with gold nanoparticles or AMG traceable quantum dots [11,14].

In the present study, we used gold nanoparticles as a model. Gold nanoparticles were considered suitable as a model for studying cellular uptake of nanoparticles because: 1) they are believed to be totally inert, i.e. have no adverse effects [15]; 2) they can be silver enhanced by AMG to visible sizes [5,9,11]. We decided to use 2 and 40 nm particles in order to test if any biodistribution patterns could be related to particle size.

The finding that both 2 and 40 nm particles accumulate overwhelmingly in the Kupffer cells of the liver is somewhat surprising. Even after IP injections, where a substantial part of the particles must be expected to pass through one or more lymph nodes packed with macrophages, AMG silver enhanced gold particles were found only in relatively few macrophages and only in few animals.

Pilot experiments in our laboratory have indicated that macrophage cultures phagocytose gold nanoparticles easily, and we therefore would have expected to constantly find gold nanoparticles in macrophages in lymph nodes draining the injection site. At this point we think that as the IP injected nanoparticles might have penetrated the peritoneum any places in the abdomen we might only by change have removed the "right" lymph node i.e. the ones draining a region where substantial amounts of nanoparticles have penetrated. However, our results prove beyond doubt that the Kupffer cells are central in the elimination process of nanoparticles that cross the epithelial barriers of the body.

The reasons why exposure to 2 nm particles resulted in a loading of only a minor part of the Kupffer cells even when given IV could be: 1) *The concentration of gold*: 1 ml of colloidal gold containing 40 nm particles contains 58.21 micrograms of pure gold, whereas 1 ml of colloidal gold with 2 nm particles contains just 12.13 micrograms of pure gold. 2) *Glomerular filtration*: Current knowledge on glomerular filtration [16,17] allows us to suggest that 2 nm gold nanoparticles are filtrated via the kidneys, while 40 nm nanoparticles definitely cannot be filtrated. This is expected drastically to influence the amount of circulating 2 nm particles. For humans the whole blood volume is filtered through the kidney within one hour, we have not been able to find the figures on mice.

The clear difference in gold nanoparticle uptake by the Kupffer cells, depending on the route of exposure, is most likely a result of a delayed presentation of the particles to the Kupffer cells after IP injections compared to IV injections. It is surprising, though, that the particles given IP are not taken up primarily by the myriads of macrophages which they must pass before reaching the liver.

We have not yet measured the time that the Kupffer cells stay in the liver after they have been loaded with nanoparticles. Such information will be important for our understanding of the nanoparticle trafficking in the mammalian organism in the time-scale before elimination.

Following IV injection, the Kupffer cells were heavily loaded after one hour, while only a fraction of the cells were lightly loaded one hour after an IP injection.

Our findings confirm almost to the point the results of Singer et al. [18] and Adlersberg and Singer [19]. These scientists performed two studies using 300 mice in each study. They exposed mice both IV and IP to both radioactive ^198^Au and nonradioactive colloidal gold nanoparticles with a diameter of 3–7 nm. Tissues were analyzed by measuring radioactivity and by light microscopy. The dose they used for histological observations was 250 micrograms, i.e. more than 4 times larger than the dose we used. Already one hour after exposure approximately 90% of the nanoparticles were accumulated in the liver while the remaining 10% accumulated in the rest of the body. Histologically, they found gold nanoparticles to be localized inside the macrophages. Accumulation of radioactive colloidal gold particles was primarily found in the liver and lymph nodes in animals and man, in the other studies using IP administration [20-22].

The finding that there was no significant difference in accumulation patterns within the three survival periods applied in our study (1, 4 and 24 hours) suggests that once gold nanoparticles have entered the blood circulation, they will either be trapped by the Kupffer cells in the liver, or if smaller than about 4–6 nm partially be filtrated into the preurine. A study performed by Heinfeld et al. [8] with 1.9 nm Au particles, supports this suggestion. In their study gold nanoparticle retention in the liver and spleen was low suggesting elimination through the kidneys. This mechanism seems to be very efficient and capable of protecting the rest of the organism from the nanoparticles.

Any cells able to pick up nanoparticles by endocytosis, i.e. pinocytosis or phagocytosis, will either take them up in lysosomes/phagocytomes, or export them out of the cell again. Pinocytosis might e.g. be an important factor in the transport of nanoparticles across the blood vessel wall. Recent studies have suggested that transcytosis system across the endothelium is caveollae-mediated process [23]. Considering that caveolae are 50 – 80 nm in size [24], 2 and 40 nm gold particles most likely pass cell barriers by transcytosis.

### Transplacental transport

Gold nanoparticles have been administered to pregnant animals before. Challier et al. [25] used 4–200 nm gold radiocolloid particles and demonstrated impermeability of rat placenta in both directions, i.e., mother-fetus and fetus-mother. Our findings support this. However, Takahashi and Matsuoka [26] exposed rats IV to 5 and 30 nm _198_Au particles and reported an insignificant transfer to the fetus, i.e. 0.018 and 0.005% for 5 and 30 nm particles, respectively. In our study, there were no traces of gold nanoparticles neither in fetus nor placenta, which suggests that those particles cannot cross the placenta barrier.

## Conclusion

Our results suggest that: (1) gold nanoparticles do not penetrate cell membranes by non-endocycytotic mechanisms, but is rather transported through cells by transcytosis (2) gold nanoparticles in the 2–40 nm interval are taken up primarily by Kupffer cells in the liver and secondarily by macrophages in the spleen and in other places; (3) gold nanoparticles do not seem to penetrate the placenta barrier; (4) the blood-brain barrier seems to protect the central nervous system from gold nanoparticles; (5) endocytosis by macrophages seems not to be the only way that the organism use to eliminate 2 nanometer gold particles. We hypothesize that part of these tiny nanoparticles are released into the urine as a result of simple filtration in the renal glomeruli.

## Methods

### Gold nanoparticles

We used 2 and 40 nm colloidal gold nanoparticles. The 2 nm gold nanoparticles solution contained 15 × 10^13 ^particles per 1 ml (12.13 micrograms) while the 40 nm gold solution contained 9 × 10^10 ^particles per 1 ml (58.21 micrograms) Both 2 nm and 40 nm gold nanoparticles were acquired from Fitzgerald Industries Inc, USA. These particles were made by citrate reduction and therefore had a negative surface charge. The gold nanoparticles were monodisperse and spherical in shape. The solution additionally contained 0.01% AuCl and traces of citrate, pH = 5.5.

### The animal model

The study was undertaken in accordance to the Danish law and the University of Aarhus guidelines for animal welfare. A total of 46 female C57BL/6 mice were used, 13 of which were pregnant. The 33 non-pregnant animals were 120 days old at a body of weight of 18 to 21 grams. They were divided into nine experimental groups and two control groups, each consisting of 3 mice. Six experimental groups were injected IV with 1 ml colloidal gold containing either 2 or 40 nm gold nanoparticles. The intravenous injections were given into the tail vein. The injections were performed slowly, over a period of no less than 5 min per injection. The animals were carefully observed during the injection procedure and the observation continued one hour after its termination. Three experimental groups were exposed IP to 1 ml colloidal gold, containing 40 nm gold nanoparticles. 6 mice served as controls: 3 of them were exposed to 1 ml saline IV, and the other 3 to 1 ml saline IP. To prevent any possible discomfort for the animals during the post exposure period, analgesia was ensured by adding buprenorphin to the drinking water. The animals were housed in plastic cages under the following conditions: 12 h light/dark cycle, 22 +/- 2°C and 50 +/-10% relative humidity. Food (Altromin No. 1314, Altromin Spezialfutterwerke, Germany) and tap water were available ad libitum. The nine experimental groups were allowed to survive for: 1, 4 and 24 hours, respectively, and the control groups 24 hours.

*The pregnant animals *were in their 16–18^th ^day of pregnancy, at a body weight of 29 – 31 grams. They were divided into two experimental groups of five animals. These groups received IV 1 ml of a solution containing 2 nm gold nanoparticles and 1 ml of a solution containing 40 nm gold nanoparticles, respectively. The remaining three pregnant animals served as controls and were exposed to 1 ml saline IV.

All mice were anesthetized with isofluran and sodium pentobarbital (50 mg/kg body weight) and transcardially perfused at 120 mm Hg with 3% buffered glutaraldehyde for 5 min (1 min of rapid flow followed by 4 min of reduced flow 5–10 ml/min). The brain, small intestine (ileum), mesenterial lymph nodes, liver, spleen, kidneys, adrenals, and ovaries were excised and placed in glutaraldehyde for 24 hours at 8°C temperature. From the pregnant mice fosters, placenta and liver from the mother animals were excised.

### Tissue processing

The organs selected for cryostat sectioning were placed in 30% sucrose until they sank to the bottom of the jar. They were frozen with carbon dioxide and cut into 30 micrometers sections on a Dittes-Duspiva Cryostat, placed on glass slides, dried and coated with gelatin.

Tissue to be embedded in Epon blocks was cut in approximately 2 × 2 × 2 mm blocks with a razorblade or scalpel and rinsed in 0.10 M phosphate buffer (pH 7.4) for 2 × 5 min, then dehydrated in graded ethanol solutions and embedded in Epon. Semithin sections (3 μm) were cut with an ultramicrotome (Leica EM UC6) and placed on glass slides before being AMG developed. After light microscopic analyses, selected sections were re-embedded on top of a blank Epon block and trimmed to include only the regions of interest. Thereafter, ultrathin sections were cut and counterstained with lead citrate and uranyl acetate before electron microscopic analysis (Philips Morgagni 268D).

#### AMG development

The AMG developer consisted of a 60 ml gum arabic solution and 10 ml sodium citrate buffer (25.5 g of citric acid · 1H_2_O + 23.5 g sodium citrate · 2H_2_0 to 100 ml distilled water). Immediately before use 15 ml reductor (0.85 g of hydroquinone dissolved in 15 ml distilled water at 40°C) and 15 ml of a solution containing silver ions (0.12 g silver lactate in 15 ml distilled water at 40°C) were added, and the AMG developer was thoroughly stirred [11]. The glass slides were put in a jar filled with the AMG developer and placed in a water bath at 26°C. The entire set-up was covered with a dark hood. During the AMG development an electric device shook the jars gently. After 60 minutes the AMG development was stopped by rinsing the slices in water and afterwards replacing the developer with a 5% sodium thiosulphate solution for 10 min (the AMG stop bath solution). The jars were then placed under gently running water for 20 minutes.

#### Post AMG treatment

The sections from the different sources were counterstained with a 0.1% aqueous toluidine blue solution (pH 4.0), dehydrated in alcohol to xylene, and ultimately embedded in DEPEX and covered with a cover glass. Black silver grains represented silver-encapsulated gold nanoparticles. All procedures and protocols have previously been described in details [11].

## Competing interests

The author(s) declare that they have no competing interests.

## Authors' contributions

ES worked on the design of the study, acquisition and interpretation of the data, and on drafting the manuscript;

HW was involved in the design, interpretation of the results and reviewed the manuscript;

MS was involved in the acquisition of the electron microscopy pictures and interpretation of the results, and reviewed the manuscript;

UV was involved in the interpretation of the results and reviewed the manuscript;

PD was involved in the acquisition of the data and reviewed the manuscript;

AL was involved in the acquisition of the data and reviewed the manuscript;

GD designed the study and drafted the manuscript.

All authors read and approved the final manuscript.
